# Cystatin C and Risk of Diabetes and the Metabolic Syndrome – Biomarker and Genotype Association Analyses

**DOI:** 10.1371/journal.pone.0155735

**Published:** 2016-05-24

**Authors:** Martin Magnusson, John Molvin, Gunnar Engström, Patrik Svensson-Färbom, Margaretha Persson, Anders Christensson, Peter Nilsson, Olle Melander

**Affiliations:** 1 Department of Clinical Sciences, Lund University, Malmö, Sweden; 2 Department of Cardiology, Skåne University Hospital, Malmö, Sweden; 3 Center of Emergency Medicine, Skåne University Hospital, Malmö, Sweden; 4 Department of Nephrology, Skåne University Hospital, Malmö, Sweden; College of Medicine, National Taiwan University, TAIWAN

## Abstract

**Background:**

We recently reported a relationship between plasma levels of cystatin C and incidence of the metabolic syndrome (MetS) among the first 2,369 subjects who participated in the re-examination study of the population-based Malmö and Diet Cancer Cardiovascular cohort (MDC-CC-re-exam). In this study we aimed to replicate these results and also investigate if cystatin C was causally associated with MetS and diabetes.

**Methods:**

We estimated the effect size of the strongest GWAS derived cystatin C SNP (major allele of rs13038305) on plasma cystatin C in the now completed MDC-CC-re-exam (n = 3,734) and thereafter examined the association between plasma cystatin C (403 cases of diabetes and 2665 controls) as well as rs13038305 (235 cases and 2425 controls) with incident diabetes. The association of rs13038305 and incident MetS (511 cases of MetS and 1980 controls) was similarly investigated in the whole MDC-CC-re-exam. We also attempted to replicate our previously shown association of cystatin C with incident MetS in subjects from the MDC-CC-re-exam (147 cases and 711 controls) that were not included in our previous report.

**Results:**

In the entire MDC-CC-re-exam, each copy of the major allele of rs13038305 was associated with approximately 0.30 standard deviation (SD) higher plasma concentration of cystatin C (β = 0.33, p = 4.2E^-28^) in age and sex adjusted analysis. Cystatin C in plasma was not associated with incident diabetes after adjustment for known diabetes risk factors (OR per 1 SD increment 0.99 (0.86–1.13), p = 0.842). In the replication cohort of MDC-CC-re-exam, the OR (95% CI) for incident MetS in subjects belonging to quartiles 1, 2, 3 and 4 of plasma cystatin C levels was 1.00 (reference), 1.21 (0.70–2.07), 1.62 (0.95–2.78) and 1.72 (1.01–2.93) (p_trend_ = 0.026) in age and sex adjusted analysis. In the entire MDC-CC-re-exam the odds ratio for incident MetS and diabetes per copy of the major rs13038305 allele was 1.13, (0.95–1.34), p = 0.160 and 1.07, 95% CI 0.89–1.30, p = 0.478, respectively.

**Conclusion:**

We were able to replicate our previously shown association between high levels of cystatin C and increased risk of future development of MetS. However, a causal involvement of cystatin C in the etiology of MetS or diabetes seems unlikely since genetic elevation of plasma cystatin C was not significantly related to incidence of these diseases.

## Introduction

Cystatin C, an established sensitive marker of glomerular filtration [1], has also been widely recognized as a strong predictor of cardiovascular disease (CVD) [2–5], this even in populations within apparently normal ranges of renal function [6, 7]. In addition, both cross-sectional and prospective studies have identified cystatin C to be associated with metabolic risk factors [8–11] as well as the incidence of the metabolic syndrome (MetS) [12] and diabetes [13, 14]. However, although these studies indicate that cystatin C might serve as a marker of disease susceptibility, cystatin C′s causal involvement and directionality of any causal effect on these diseases is yet to be proven. The biological mechanisms behind a possible influence of the cystatin C metabolism on CVD, MetS and diabetes risk are unclear.

In this study we performed a re-examination of the population-based Malmö Diet and Cancer (MDC) study cardiovascular cohort (MDC-CC) 17 years after the baseline examination (MDC-CC-re-exam), and tested the hypothesis that a high baseline plasma level of cystatin C was associated with the development of the MetS and diabetes during long-term follow-up. To test whether any association between cystatin C and the MetS was driven by any individual components of MetS, baseline plasma level of cystatin C was related to the incidence of each of the five different components. Furthermore, since genome-wide association studies (GWAS) for genetic determinants of cystatin C have identified the strongest signal of the genome associating with variation of plasma concentration of cystatin C at the cystatin C locus on chromosome 20, represented by the single nucleotide polymorphism (SNP) rs13038305 [15], we set out to investigate if there was a causal association between cystatin C and glucometabolic disease by relating rs13038305 (major allele) to the development of MetS and diabetes.

## Material and Methods

### Study Sample

**Fig 1:**
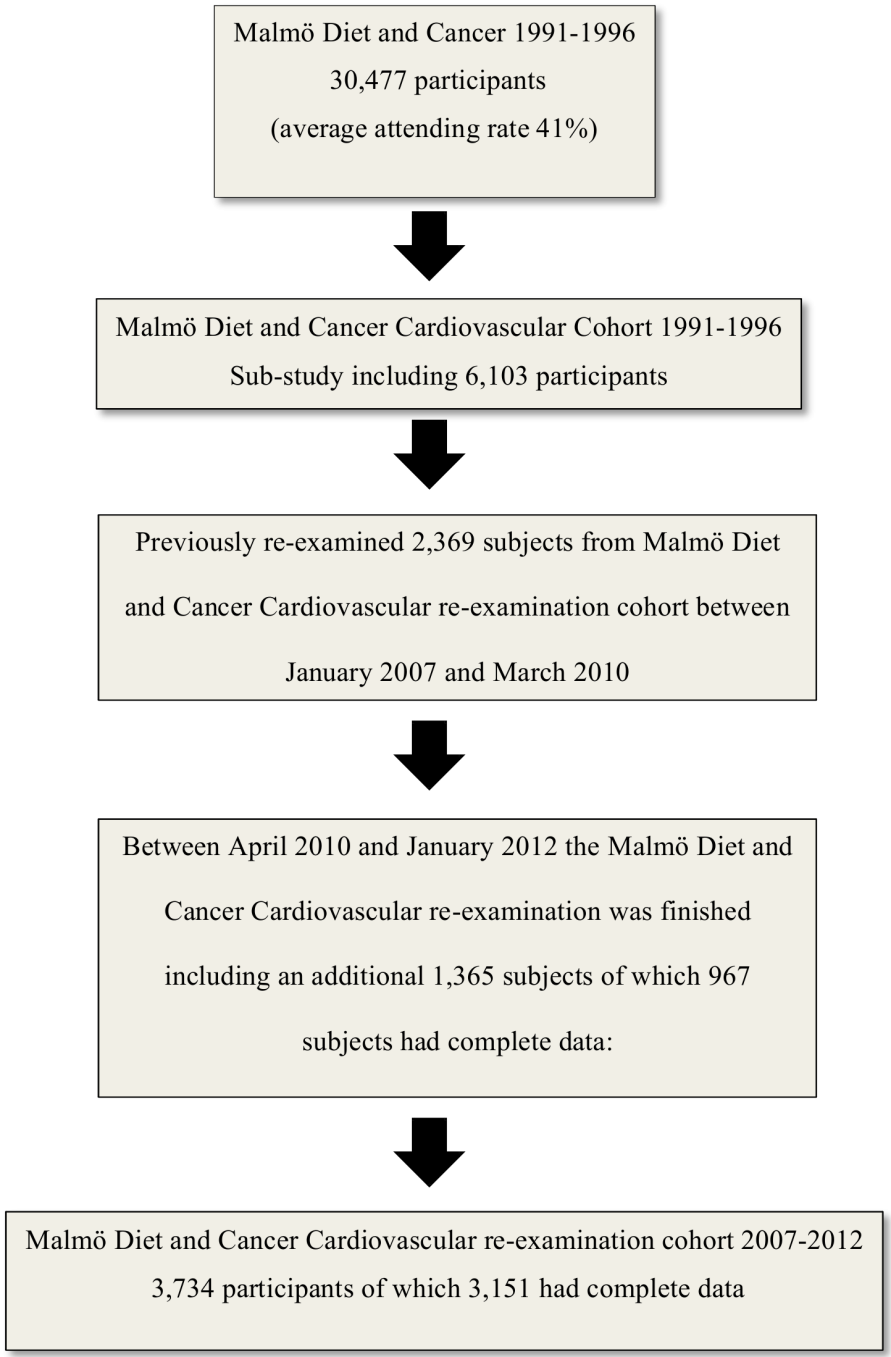
Flow chart describing the study-populations.

The MDC study is a population-based study that enrolled 28,449 individuals between 1991 and 1996 [16]. During the years of 1991–1994 a random sample of the study subjects were selected to study the epidemiology of carotid artery disease (n = 6103) and this sample is referred to as the MDC-CC. Fasting plasma samples were obtained in 5,405 subjects in the MDC-CC. [17] We have previously published results from the at that time on-going re-examination study of the MDC-CC, where we re-examined 2,369 subjects of these 5,405 subjects between January 2007 and March 2010. This study cohort was used to investigate cystatin C association with MetS and these data have been reported for previously [12]. Between April 2010 and January 2012 this re-examination was finished including an additional 1,365 subjects again using the same protocol similar to that applied at the baseline exam but with addition of a 75 gram oral glucose tolerance test with measurement of plasma glucose fasted at time 0 min and after 120 min (Fig 1). Of them, 967 subjects had complete data of all covariates (age, sex, waist circumference (waist), systolic blood pressure (SBP), diastolic blood pressure (DBP), high density lipoprotein (HDL), triglycerides (TG), anti hypertensive treatment (AHT) and cystatin C measurements in plasma at baseline) and were also successfully genotyped for rs13038305 and these subjects (MDC-CC-re-examination-replication-cohort) were used as a replication cohort to validate our previous findings showing that increased plasma levels of cystatin C is associated with increased risk of MetS.

Of the 967 subjects (MDC-CC-re-examination-replication-cohort), 109 subjects had prevalent MetS at the baseline examination, resulting in a total of 858 subjects (147 cases and 711 controls) for the analysis of incident MetS. In analyses of the incidence of the five subcomponents of the MetS, patients with of each of the five separate MetS criteria at the baseline examination were excluded separately, resulting in 878 subjects (285 cases and 593 controls) with complete data for the analysis of incident large waist circumference (abdominal obesity), 745 subjects (82 cases and 663 controls) for reduced HDL levels, 807 subjects (25 cases and 782 controls) for elevated TG levels, 927 subjects (295 cases and 632 controls) for elevated glucose levels and only 350 subjects (169 cases and 181 controls) for incidence of elevated blood pressure (this was due to the high prevalence of elevated blood pressure according to National Cholesterol Education Program (NCEP) criteria at the baseline examination). The mean follow-up time for all these subgroups was 18±1 years.

The whole MDC-CC re-examination cohort (re-examined from January 2007 to January 2012) consisted of 3,734 subjects, (mean follow-up time was 17±1 years) (Fig 1) and, of these 3,151 had complete data of all covariates (age, sex, waist, SBP, DBP, HDL, TG, AHT and had valid cystatin C measurements in plasma).

Since we had not previously examined plasma cystatin C′s association with incident and prevalent diabetes, this cohort of 3,151 subjects was used for the study of such possible associations. Of the 3,151 subjects, 83 had prevalent diabetes at the baseline examination, resulting in a total of 3,068 subjects (403 cases and 2,665 controls) for the analysis of incident diabetes. The mean follow-up time was 17±1 years. We also investigated plasma cystatin C association with incident MetS in the entire MDC-re-examination cohort excluding patients with prevalent MetS at baseline (n = 442), leaving subjects 2709 subjects (562 incident MetS cases and 2147 controls). The mean follow-up time was 17±1 years. rs13038305 was successfully genotyped in 2,900 of these subjects with full data of all covariates. Of these 409 had prevalent MetS at baseline leaving 2491 subjects (511 cases and 1980 controls) for the study of the major allele of rs13038305 association with incident MetS. The mean follow-up time was 17±1 years. Finally of the 2,900 successfully genotyped subjects with full data of all covariates, 79 had prevalent diabetes at baseline leaving 2821 subjects (366 cases and 2455 controls) for the study the major allele of rs13038305 association of incident diabetes with a mean follow up time of 17±1 years. All participants provided written informed consent, and the ethical committee at Lund University, Lund, Sweden approved the study.

### Clinical Assessment

Clinical characteristics of the MDC-CC-re-examination-replication-cohort at the baseline examination are shown in Table 1 and that of the entire MDC-CC-re-examination-cohort in Table A in S1 File. Participants underwent a physical examination with standardized medical history and laboratory assessment. Blood pressure was obtained after resting for 10 min in the supine position using the Omron^®^ automatic device. We calculated body mass index (BMI) as weight in kilograms divided by the square of the height in meters. Hypertension was defined as SBP ≥140 mmHg or diastolic blood pressure ≥90 mmHg, or use of AHT. The examination protocol performed by a trained research assistant included measurement of maximal waist circumference, fasting plasma glucose (FPG), fasting plasma insulin, fasting TGs, cholesterol, low density lipoprotein (LDL) cholesterol and high density lipoprotein (HDL). Medical history and current medication were retrieved by a self-administered questionnaire.

**Table 1:** Baseline characteristics of the study participants in the MDC-CC-re-examination-replication-cohort by prevalence of the metabolic syndrome.

*n*	Subjects with prevalent MetS 109	Subjects without MetS 858
Age (years)	55.6±5.6	55.1±5.3
Sex (% women)	58.7	65.1
Current smoker (%)	22.0	22.7
BMI (kg/m^2^)	29.1±4.6	24.7±3.2
Waist circumference (cm)	93.7±11.9	79.7±10.7
Systolic BP (mmHg)	144.0±13.8	136.0±17.1
Diastolic BP (mmHg)	90.7±8.6	84.9±8.7
Antihypertensive medication* (%)	24.8	9.0
Hypertension (%)	68.8	36.9
F-glucose (mmol/L)	5.7±1.5	4.9±0.8
T-cholesterol (mmol/L)	6.5±1.0	6.0±1.1
LDL (mmol/L)	4.3±0.9	4.0±1.0
TGs (mmol/L)	2.3±1.0	1.1±0.5
HDL (mmol/L)	1.1±0.2	1.5±0.4
Cystatin C (mg/L)	0.8±0.1	0.7±0.1
Diabetes (%)	10.1	1.5

BMI, body mass index; BP, blood pressure; F, fasting; T, total; TGs, triglycerides; MetS, metabolic syndrome. Values are mean (± SD) or frequency in percentage.

* Hypertension is defined as blood pressure above 140/90 mmHg or the use of antihypertensive medication.

### Laboratory Assays

All analyses in plasma and whole blood were performed using overnight fasting samples. Analyses of fasting plasma lipids, FBG (baseline examination) and FPG (re-examination) were carried out at the Department of Clinical Chemistry, Skåne University Hospital in Malmö, which is part of a national standardization and quality control system. Levels of plasma cystatin C were measured using a particle-enhanced immunonephelometric assay (N Latex Cystatin; Dade Behring, Deerfield, Illinois) and presented in mg/L. The assay has an intraindividual coefficient of variation of 7.7% percent. The range of detection of the assay is 0.195 to 7.330 mg per liter, with the reference range for young, healthy persons reported as 0.53 to 0.95 mg per liter. eGFR was assessed with the formulae of Cockroft-Gault creatinine clearance (mL/min/1.73 m2) = (140-age) x weight in kg x 1.23 / P-creatinine (x 0.85 if female) (CG) [18].

### Genotyping

DNA was extracted from frozen granulocytes or buffy coats with the use of QIAamp-96 spin blood kits (QIAGEN, Stockholm, Sweden) at the DNA extraction facility supported by SWEGENE. We successfully genotyped the plasma cystatin C associated SNP rs13038305 at the cystatin C locus on chromosome 20 in 3,105 subjects of the MDC-CC-re-exam using TaqMan^®^ Assay by Design primers and probes, with a real-time PCR assay using the ViiA7 equipment (Applied Biosystems, Foster City, CA, USA) according to the manufacturer’s instructions [19]. As this cohort has been analyzed with a GWAS (HumanOmniExpressBeadChip and iScan system (Illumina, San Diego, CA, USA)) we were able to check for concordance between the TaqMan based and GWAS-based method, which showed more than 99.5% concordance.

### Definition of MetS

The MetS was defined according to the Third Report of the NCEP (NCEP-ATP-III) [20]. We intentionally did not test the revised NCEP-ATP III (i.e. AHA/NHLBI) definition as most recent studies have applied the unrevised version, and in fact the difference between the two definitions is minimal (correction of FPG by 0.5 mmol/L). In addition to the blood pressure threshold of ≥130/85 mmHg, we regarded subjects on AHT as fulfilling the criteria for hypertension.

### Definition of Diabetes

Prevalent diabetes was defined as FBG ≥6.1 mmol/L, a history of physician diagnosed diabetes mellitus or treatment with antidiabetic medication at the time of the baseline examination. Incident diabetes was defined as fasting plasma glucose of at least 7.0 mmol/liter or a 2-h plasma glucose value of at least 11.0 mmol/liter during the OGTT at the reexamination or history of physician diagnosis of diabetes or initiation of antidiabetic medication any time after the baseline exam.

### Statistical Analysis

A genome wide association study (GWAS) was performed for plasma levels of cystatin C using additive genetic models of inheritance and linear regression adjusting for age and sex. We used logistic regression models to calculate the odds ratio (OR) of prevalent MetS and diabetes at the baseline examination adjusting for age and sex and the OR of incident MetS and diabetes (after excluding patients with the MetS and diabetes, respectively at the baseline examination). OR were calculated per 1 SD increment of cystatin C and per copy of the major C allele of rs13038305 in models adjusted for age and sex (model 1) and also in multivariate analysis adjusted for age, sex, waist, AHT, SBP, TGs, HDL and FBG (model 2). Furthermore, in order to test whether any relationship between cystatin C and MetS and diabetes were linear, incidence and prevalence of diabetes and MetS were studied across quartiles of cystatin C levels. A multivariate logistic regression model (adjusted model 1) was used to assess incidence of the five individual components of the MetS (after excluding patients with each of the five separate MetS criteria at the baseline examination). The OR for the individual MetS criteria were calculated per 1 SD increment of cystatin C and per copy of the major rs13038305 allele. In order to study if other estimates of renal function (e.g. glomerular filtration rate as estimated by eGFR-CG) would influence the association of between cystatin C and Mets and diabetes, eGFR-CG was entered on top of all other variable in model 2 in the multivariate logistic regression analysis. Because we did not find any significant interaction between gender and cystatin C levels with respect to incidence of the MetS (*p* = 0.234), or diabetes (*p* = 0.452), in the fully adjusted model 2, sex-specific analyses was not performed. All analyses were performed using SPSS Windows version 22.0 and a two-tailed *P* value of <0.05 was considered statistically significant.

## Results

Characteristics of the study participants with and without MetS in the MDC-CC re-examination replication cohort are shown in Table 1 and characteristics of the study participants with and without diabetes in the MDC-CC re-examination cohort are shown in Table A in S1 File.

### rs13038305 in Relation to Cystatin C

At baseline each copy of the major allele of rs13038305 was associated with a highly significant approximately 0.30 standard deviation (SD) higher plasma concentration of cystatin C both in model 1 (β = 0.33, p = 4.2E-28) and model 2 (β = 0.33, p = 1.7E-29).

### Cystatin C and Relation to Prevalent MetS and Diabetes–Cross-Sectional Analysis

At baseline, each 1 SD increment of cystatin C was significantly associated with an increased prevalence of MetS (adjusted for age and sex) (OR per 1 SD increment 1.36 (1.13–1.65), p = 0.001) (Table B in S1 File). Prevalence of MetS at the baseline examination increased significantly in a linear manner across quartiles of cystatin C at baseline (p for trend = 0.001) (Table B in S1 File). Furthermore, in cross-sectional analyses adjusted for age and sex, prevalence of increased waist circumference, decreased HDL and increased TG, according to the limits defined by the NCEP-ATP-III guidelines, were all significantly associated with increased cystatin C (Table C in S1 File). However, cystatin C was not significantly associated with prevalence of diabetes adjusted for age and sex (OR per 1 SD increment 0.90 (0.71–1.14), p = 0.384).

### Cystatin C and Relation to Incident MetS and Diabetes–Prospective Analysis

In the prospective analyses in the MDC re-examination-replication cohort of the patients without the MetS at baseline, 147 developed MetS during follow-up. The increased MetS risk across quartiles of cystatin C levels was significant in the age- and sex-adjusted model (Table 2) but not in the fully adjusted regression according to model 2 (Table 2).

**Table 2:** Cystatin C and risk of future metabolic syndrome in the MDC-CC-re-examination-replication-cohort.

Cystatin C		
Model 1		Model 2
**Cystatin C as a continuous variable—**		
**Per SD increment**	1.17 (0.99–1.39)	0.98 (0.80–1.19)
***P***	0.072	0.812
**Cystatin C as a categorical variable—**		
**<Q1**	1.0 (Referent)	1.0 (Referent)
**Q1–median**	1.21 (0.70–2.07)	0.90 (0.50–1.63)
**Median–Q3**	1.62 (0.95–2.78)	1.09 (0.60–1.96)
**>Q3**	1.72 (1.01–2.93)	1.02 (0.56–1.85)
***P for trend***	0.026	0.790

Values are odds ratios (95% confidence intervals) for incident MetS from logistic regression analyses. Model 1 is adjusted for age and sex; model 2 is adjusted for age, sex, systolic blood pressure, antihypertensive treatment, waist circumference, plasma levels of triglycerides, fasting whole-blood glucose and HDL at baseline. *n* = 858 with 147 MetS cases and 711 controls.

In the prospective analyses (age and sex adjusted) of the five individual components of MetS, baseline cystatin C was significantly related to incident abdominal obesity and incident elevated TG levels but not with incident hypertension, reduced HDL levels and incident hyperglycemia (Table 3). Due to the small sample size in the MDC re-examination-replication cohort and many variables to be considered in the fully adjusted model we also investigated if plasma levels of cystatin C were associated with incident MetS in the whole MDC-CC-re-examination cohort (562 MetS cases and 2147 controls). The increased MetS risk across quartiles of cystatin C levels was significant in the fully adjusted model 2 (Table 4). In the logistic regression regarding risk of developing incident MetS in the whole MDC-re-examination cohort when adding eGFR-CG on top of model 2 showed that each 1 SD change of eGFR-CG was highly significantly associated with incident MetS (OR 1.41 95% CI (1.21–1.64), p = 1.00x10^-5^, and the relation of cystatin C with incident MetS was (OR 1.14 (1.02–1.27), p = 0.019).

**Table 3:** Cystatin C and rs13038305 and incidence of the individual components of the metabolic syndrome in the MDC-CC-re-examination-replication-cohort.

Cystatin C		
	Odds ratio (95% confidence intervals)	*P* value
*Component*		
**Large waist circumference (abdominal obesity)**	1.23 (1.06–1.44)	0.007
**Elevated triglycerides**	1.53 (1.07–2.18)	0.021
**Reduced HDL**	1.01 (0.80–1.28)	0.941
**Elevated fasting glucose**	1.07 (0.93–1.24)	0.347
**Elevated blood pressure**	0.86 (0.68–1.09)	0.213
**rs13038305**		
**Large waist circumference (abdominal obesity)**	0.99 (0.78–1.26)	0.941
**Elevated triglycerides**	1.39 (0.65–2.94)	0.395
**Reduced HDL**	1.16 (0.78–1.72)	0.476
**Elevated fasting glucose**	1.05 (0.82–1.33)	0.717
**Elevated blood pressure**	0.76 (0.53–1.09)	0.139

Components of the metabolic syndrome are defined in the text. Values are odds ratios (95% confidence intervals) per 1 SD increment of cystatin C for incident components of the metabolic syndrome from logistic regression analyses. All models were adjusted for age and sex at baseline

**Table 4:** Cystatin C and risk of future metabolic syndrome in the MDC-CC-re-examination-cohort.

Cystatin C	
**Cystatin C as a continuous variable**	
**Per SD increment**	1.09 (0.98–1.21)
***P***	0.115
**Cystatin C as a categorical variable**	
**<Q1**	1.0 (Referent)
**Q1–median**	1.01 (0.74–1.38)
**Median–Q3**	1.12 (0.83–1.53)
**>Q3**	1.40 (1.03–1.90)
***P for trend***	0.023

Values are odds ratios (95% confidence intervals) for incident MetS from logistic regression analyses. adjusted for age, sex, systolic blood pressure, antihypertensive treatment, waist circumference, plasma levels of triglycerides, fasting whole-blood glucose and HDL at baseline. *n* = 2709 with 562 MetS cases and 2147 controls.

During follow up 403 patients developed new onset diabetes and each 1 SD increment of cystatin C was significantly associated with an increased risk of incident diabetes adjusted for model 1 (OR per 1 SD increment 1.14 (1.03–1.27), p = 0.014) but this association was totally attenuated in the fully adjusted according to model 2 (0.99 (0.86–1.13), p = 0.842) (Table D in S1 File). As for the analysis of baseline values of cystatin C and incident diabetes adding eGFR-CG on top of model 2 each 1 SD change of GFR showed no significant association with incident diabetes (OR 0.95 (0.80–1.14), p = 0.601 and baseline cystatin C remained not associated with incident diabetes (OR 0.99 (0.86–1.13), p = 0.835.

### rs13038305 and MetS

The major allele of rs13038305 was not associated with prevalent MetS after adjustment for age and sex (OR 1.00, 95% CI 0.84–1.20, p = 0.990). Furthermore, the major allele of rs13038305 was not associated with prevalence of the individual components of the MetS (Table C in S1 File). In addition, no significant associations were seen for rs13038305 and incident MetS (OR 1.13, 95% CI 0.95–1.34, p = 0.160) in age and sex adjusted logistic regression analysis. As for the cross-sectional analysis, in the prospective analysis no significant associations were seen for the incidence of the five different MetS components and rs13038305 after adjustment for model 2 (Table 3).

Finally, no significant associations were seen for rs13038305 and all MetS cases (both prevalent and incident) after adjustment for age and sex (OR 1.08, 95% CI 0.95–1.24, p = 0.242)

### rs13038305 and Diabetes

The rs13038305 polymorphism was not associated with prevalent diabetes after adjustment for age and sex (OR 0.78, 95% CI 0.55–1.12, p = 0.179). In addition, no significant associations were seen for rs13038305 and incident diabetes after adjustment for age and sex (OR 1.07, 95% CI 0.89–1.30, p = 0.478). Finally, no significant associations were seen for rs13038305 and all diabetes cases (both prevalent and incident) after adjustment for age and sex (OR 1.01, 95% CI 0.85–1.20, p = 0.891).

## Discussion

The key findings of our study are that although we were able to replicate our previous results that high plasma levels of cystatin C predicts new on-set of MetS and also increased risk of new on-set diabetes in age and sex adjusted analysis, these association were completely attenuated when known diabetes and MetS risk factors were entered as covariates in the multiple regression analysis. Furthermore, even if our findings, adjusted for known MetS risk factors in the entire MDC-CC-reexamination cohort, indicates that high levels of cystatin C measured in plasma might serve as a predictor of future MetS, a causal involvement of cystatin C in the etiology of MetS or indeed diabetes seems unlikely since genetic elevation of plasma cystatin C (e.g. major allele C of rs13038305) was not related to altered risk of these diseases.

There has been an intense discussion regarding the biological mechanism behind the possible dysmetabolic and CVD increasing effects of elevated cystatin C levels over the last decade, although the exact mechanisms behind the observed correlations have not been comprehensively clarified. The most common explanation behind high cystatin C levels and poor cardiovascular or metabolic outcome is attributed to impaired renal function, that is, because cystatin C is able to detect minor impairments of renal function in the normal GFR range compared to creatinine, even minor impairment of renal function may have clinical significance thus explaining the associations between elevated plasma levels of cystatin C and increased risk of disease in prior studies [3] [14].

In a recently published paper by Shlipak (senior author) in 2014, where genetically lowered cystatin C (here the minor T allele of rs13038305) was tested for possible associations with incident CVD in a population of 14,645 individuals, no significant associations were seen for incident CVD, OR, (95% CI), 0.98 (0.92–1.06), p = 0.7 nor total mortality 0.98 (0.92–1.03), p = 0.4 arguing against a direct cystatin C mediated effect on CVD/mortality-risk [21]. In addition, a recent publication by Svensson-Farbom and co-workers reported that genetic elevation of plasma cystatin C was not related to altered risk of coronary artery disease (CAD), supporting the notion that there is no causal relationship between plasma cystatin C and CAD. Rather, the association between cystatin C and CAD appeared to be due to the association of renal dysfunction and CAD [22]. Also, we could see that kidney function as estimated by eGFR-CG was highly significantly associated with incident MetS, and further that cystatin C′s association with incident MetS remained significant when eGFR was taking into account in the multivariate logistic regression analysis, data that further stresses the notion that cystatin C is a reflector of increased risk of MetS due to renal dysfunction.

There are however studies that indicates that rs13038305 elevate plasma cystatin C independetly of renal function and other CVD and diabetes risk factors [15] and one might speculate that this renal function independency of the genetic effect could serve as an explanation behind the lack of effect of rs13038305 on incident metabolic and cardiovascular disease.

Our study is to the best of our knowledge the first to examine the effects of genetic elevated cystatin C (here the major C allele of rs13038305) on incident diabetes and MetS. Here rs13038305 showed no significant associations with diabetes nor MetS development, and these data, together with the findings by Shlipak [21] and Svensson-Farbom [22], further emphasize the notion that cystatin C may not be causally involved in etiology of metabolic or cardiovascular disease. These results are possibly of great value since causality assessment of biomarkers of disease may provide guidance on whether or not drug development targeted at the biomarker in question is worthy to pursue, and thus this might not be the case for cystatin C.

Strengths and limitations of this study should be considered. We included a large population sample and the definition of new-onset diabetes and MetS was based on clinical re-examination. Through clinical re-examination we were able to retrieve the great majority of incident cases of diabetes and MetS. However, we were limited because rs13038305 explained only about 14 percentage of cystatin C variation (r^2^ = 0.142), which limits the causal estimations of cystatin C, however this is to be compared with the work by Shlipak (senior author) in which each copy of the minor T allele of rs13038305 was associated with 6% lower cystatin C levels (*P* = 4.1×10^−113^) [21]. Of note, individuals included in the current study were a minority of the total study population. As all subjects were survivors, they are likely to represent a healthier cohort than the entire MDC-CC. Finally; our study results may not be generalizable to all populations as the study participants were mainly individuals of Swedish descent.

## Conclusion

In conclusion, our findings show that cystatin C measured in plasma might serve as a predictor of future MetS but not diabetes. A causal involvement of cystatin C in the etiology of MetS or diabetes seems unlikely since genetic elevation of plasma cystatin C was not significantly related to these diseases.

## Supporting Information

S1 File Tables A-D. (DOCX)

## References

[1] Dharnidharka VR, Kwon C, Stevens G. Serum cystatin C is superior to serum creatinine as a marker of kidney function: a meta-analysis. American journal of kidney diseases: the official journal of the National Kidney Foundation. 2002;40(2):221–6. Epub 2002/07/31. 10.1053/ajkd.2002.34487 . 12148093

[2] Hoke M, Amighi J, Mlekusch W, Schlager O, Exner M, Sabeti S, et al Cystatin C and the risk for cardiovascular events in patients with asymptomatic carotid atherosclerosis. Stroke; a journal of cerebral circulation. 2010;41(4):674–9. 10.1161/STROKEAHA.109.573162 . 20150544

[3] Shlipak MG, Sarnak MJ, Katz R, Fried LF, Seliger SL, Newman AB, et al Cystatin C and the risk of death and cardiovascular events among elderly persons. The New England journal of medicine. 2005;352(20):2049–60. Epub 2005/05/20. 352/20/2049 [pii] 10.1056/NEJMoa043161 . 15901858

[4] Shlipak MG, Wassel Fyr CL, Chertow GM, Harris TB, Kritchevsky SB, Tylavsky FA, et al Cystatin C and mortality risk in the elderly: the health, aging, and body composition study. Journal of the American Society of Nephrology: JASN. 2006;17(1):254–61. Epub 2005/11/04. ASN.2005050545 [pii] 10.1681/ASN.2005050545 . 16267155

[5] Taglieri N, Fernandez-Berges DJ, Koenig W, Consuegra-Sanchez L, Fernandez JM, Robles NR, et al Plasma cystatin C for prediction of 1-year cardiac events in Mediterranean patients with non-ST elevation acute coronary syndrome. Atherosclerosis. 2010;209(1):300–5. 10.1016/j.atherosclerosis.2009.09.022 . 19819453

[6] Ix JH, Shlipak MG, Chertow GM, Whooley MA. Association of cystatin C with mortality, cardiovascular events, and incident heart failure among persons with coronary heart disease: data from the Heart and Soul Study. Circulation. 2007;115(2):173–9. 10.1161/CIRCULATIONAHA.106.644286 17190862 PMC2771187

[7] Loew M, Hoffmann MM, Koenig W, Brenner H, Rothenbacher D. Genotype and plasma concentration of cystatin C in patients with coronary heart disease and risk for secondary cardiovascular events. Arteriosclerosis, thrombosis, and vascular biology. 2005;25(7):1470–4. 10.1161/01.ATV.0000168416.74206.62 . 15860739

[8] Lee JG, Lee S, Kim YJ, Jin HK, Cho BM, Jeong DW, et al Multiple biomarkers and their relative contributions to identifying metabolic syndrome. Clin Chim Acta. 2009;408(1–2):50–5. Epub 2009/07/23. S0009-8981(09)00398-2 [pii] 10.1016/j.cca.2009.07.006 . 19622349

[9] Servais A, Giral P, Bernard M, Bruckert E, Deray G, Isnard Bagnis C. Is serum cystatin-C a reliable marker for metabolic syndrome? The American journal of medicine. 2008;121(5):426–32. Epub 2008/05/06. S0002-9343(08)00150-2 [pii] 10.1016/j.amjmed.2008.01.040 . 18456039

[10] Surendar J, Indulekha K, Aravindhan V, Ganesan A, Mohan V. Association of cystatin-C with metabolic syndrome in normal glucose-tolerant subjects (CURES-97). Diabetes Technol Ther. 2010;12(11):907–12. Epub 2010/10/01. 10.1089/dia.2010.0077 . 20879967

[11] Vigil L, Lopez M, Condes E, Varela M, Lorence D, Garcia-Carretero R, et al Cystatin C is associated with the metabolic syndrome and other cardiovascular risk factors in a hypertensive population. J Am Soc Hypertens. 2009;3(3):201–9. Epub 2010/04/23. S1933-1711(09)00002-3 [pii] 10.1016/j.jash.2009.01.002 . 20409960

[12] Magnusson M, Hedblad B, Engstrom G, Persson M, Nilsson P, Melander O. High levels of cystatin C predict the metabolic syndrome: the prospective Malmo Diet and Cancer Study. Journal of internal medicine. 2013;274(2):192–9. 10.1111/joim.12051 . 23414447

[13] Reutens AT, Bonnet F, Lantieri O, Roussel R, Balkau B, Epidemiological Study on the Insulin Resistance Syndrome Study G. The association between cystatin C and incident type 2 diabetes is related to central adiposity. Nephrology, dialysis, transplantation: official publication of the European Dialysis and Transplant Association—European Renal Association. 2013;28(7):1820–9. 10.1093/ndt/gfs561 . 23291367

[14] Sahakyan K, Lee KE, Shankar A, Klein R. Serum cystatin C and the incidence of type 2 diabetes mellitus. Diabetologia. 2011;54(6):1335–40. 10.1007/s00125-011-2096-6 21380596 PMC3290654

[15] Kottgen A, Glazer NL, Dehghan A, Hwang SJ, Katz R, Li M, et al Multiple loci associated with indices of renal function and chronic kidney disease. Nature genetics. 2009;41(6):712–7. 10.1038/ng.377 19430482 PMC3039280

[16] Minisymposium: The Malmo Diet and Cancer Study. Design, biological bank and biomarker programme. 23 October 1991, Malmo, Sweden. Journal of internal medicine. 1993;233(1):39–79. Epub 1993/01/01. . 8094092 10.1111/j.1365-2796.1993.tb00645.x

[17] Persson M, Hedblad B, Nelson JJ, Berglund G. Elevated Lp-PLA2 levels add prognostic information to the metabolic syndrome on incidence of cardiovascular events among middle-aged nondiabetic subjects. Arterioscler Thromb Vasc Biol. 2007;27(6):1411–6. . 17431184 10.1161/ATVBAHA.107.142679

[18] Cockcroft DW, Gault MH. Prediction of creatinine clearance from serum creatinine. Nephron. 1976;16(1):31–41. . 1244564 10.1159/000180580

[19] McGuigan FE, Ralston SH. Single nucleotide polymorphism detection: allelic discrimination using TaqMan. Psychiatric genetics. 2002;12(3):133–6. . 12218656 10.1097/00041444-200209000-00003

[20] Third Report of the National Cholesterol Education Program (NCEP) Expert Panel on Detection, Evaluation, and Treatment of High Blood Cholesterol in Adults (Adult Treatment Panel III) final report. Circulation. 2002;106(25):3143–421. Epub 2002/12/18. . 12485966

[21] O'Seaghdha CM, Tin A, Yang Q, Katz R, Liu Y, Harris T, et al Association of a cystatin C gene variant with cystatin C levels, CKD, and risk of incident cardiovascular disease and mortality. American journal of kidney diseases: the official journal of the National Kidney Foundation. 2014;63(1):16–22. 10.1053/j.ajkd.2013.06.015 23932088 PMC3872167

[22] Svensson-Farbom P, Almgren P, Hedblad B, Engstrom G, Persson M, Christensson A, et al Cystatin C Is Not Causally Related to Coronary Artery Disease. PloS one. 2015;10(6):e0129269 10.1371/journal.pone.0129269 26057752 PMC4461168

