# Supplementary material for: Cystatin C and Risk of Diabetes and the Metabolic Syndrome – Biomarker and Genotype Association Analyses
Source: PLoS One. 2016 May 24;11(5):e0155735. doi: 10.1371/journal.pone.0155735 (PMC4878806; doi:10.1371/journal.pone.0155735)
Supplement: S1 File — (DOCX) [file pone.0155735.s001.docx]

**Table A.** Baseline characteristics of the study participants in the MDC-CC-re-examination-cohort by prevalence of the diabetes.

|  | **Subjects with prevalent Diabetes** | **Subjects without Diabetes** |
| --- | --- | --- |
| *n* | 83 | 3,068 |
| Age (years) | 58.2±5.2 | 56.4±5.7 |
| Sex (% women) | 44.6 | 60.8 |
| Current smoker (%) | 6.0 | 22.3 |
| BMI (kg/m^2^) | 28.0±4.7 | 25.3±3.6 |
| Waist circumference (cm) | 91.6±13.6 | 82.2±12.1 |
| Systolic BP (mmHg) | 146.3±19.2 | 138.7±17.8 |
| Diastolic BP (mmHg) | 88.6±9.8 | 86.1±9.0 |
| Antihypertensive medication* (%) | 36.1 | 13.6 |
| Hypertension (%) | 62.7 | 45.2 |
| F-glucose (mmol/L) | 8.0±3.4 | 4.9±0.8 |
| T-cholesterol (mmol/L) | 6.2±1.0 | 6.1±1.1 |
| LDL (mmol/L) | 4.2±0.9 | 4.1±1.0 |
| TGs (mmol/L) | 1.6±1.1 | 1.3±0.7 |
| HDL (mmol/L) | 1.3±0.4 | 1.4±0.4 |
| Cystatin C (mg/L) | 0.8±0.1 | 0.8±0.1 |

BMI, body mass index; BP, blood pressure; F, fasting; T, total; TGs, triglycerides; MetS, metabolic syndrome. Values are mean (± SD) or frequency in percentage. * Hypertenison defined as bloodpresssure above 140/90 mmHg or the use of antihypertensive medication.

**Table B.** Cystatin C and risk of prevalent metabolic syndrome in the MDC-CC-re-examination-replication-cohort

| **Cystatin C** | |  |  |
| --- | --- | --- | --- |
| **Cystatin C as a continuous variable** | | |  |
| **Per SD increment** | 1.36 (1.13–1.65) |  |  |
| ***P*** | 0.001 |  |  |
| **Cystatin C as a categorical variable** | | |  |
| **<Q1** | 1.0 (Reference) |  |  |
| **Q2–median** | 2.21 (1.09–4.46) |  |  |
| **Median–Q3** | 2.24 (1.10–4.54) |  |  |
| **>Q4** | 3.44 (1.72–6.87) |  |  |
| ***P for trend*** | 0.001 |  |  |

Values are odds ratios (95% confidence intervals) for prevalent MetS from logistic regression analyses. All models are adjusted for age and sex. *n*=967 with 109 MetS cases and 858 controls.

**Table C.** Cystatin C and rs13038305 and prevalence of the individual components of the metabolic syndrome at baseline in the MDC-CC-re-examination-replication-cohort

| **Cystatin C** | | |
| --- | --- | --- |
|  | **Odds ratio (95% confidence intervals)** | ***P* value** |
| *Component* | | |
| **Large waist circumference (abdominal obesity)** | 1.36 (1.11–1.67) | 0.003 |
| **Elevated triglycerides** | 1.34 (1.13–1.59) | 0.001 |
| **Reduced HDL** | 1.37 (1.17–1.61) | <0.001 |
| **Elevated fasting glucose** | 1.00 (0.70–1.42) | 0.999 |
| **Elevated blood pressure** | 0.93 (0.81–1.07) | 0.327 |
| **rs13038305** | | |
| **Large waist circumference (abdominal obesity)** | 1.08 (0.88-1.32) | 0.460 |
| **Elevated triglycerides** | 0.95 (0.81-1.11) | 0.488 |
| **Reduced HDL** | 0.99 (0.86-1.14) | 0.837 |
| **Elevated fasting glucose** | 0.93 (0.70-1.24) | 0.621 |
| **Elevated blood pressure** | 0.97 (0.85-1.11) | 0.689 |

Components of the metabolic syndrome are defined in the text. Values are odds ratios (95% confidence intervals) per 1 SD increment of cystatin C for incident components of the metabolic syndrome from logistic regression analyses. All models were adjusted for age and sex.

**Table D**. Cystatin C and risk of future diabetes

| **Cystatin C**    **Model 1** | | **Model 2** |  |  |
| --- | --- | --- | --- | --- |
| **Cystatin C as a continuous variable** | |  | |  |
| **Per SD increment** | 1.14 (1.03–1.27) | 0.99 (0.86–1.13) |  |  |
| ***P*** | 0.014 | 0.842 |  |  |
| **Cystatin C as a categorical variable** | |  | |  |
| **<Q1** | 1.0 (Referent) | 1.0 (Referent) |  |  |
| **Q1–median** | 1.13 (0.81–1.58) | 1.04 (0.71–1.54) |  |  |
| **Median–Q3** | 1.30 (0.95–1.79) | 1.04 (0.70–1.52) |  |  |
| **>Q3** | 1.56 (1.13–2.16) | 1.01 (0.68–1.51) |  |  |
| ***P for trend*** | 0.004 | 0.981 |  |  |

Values are odds ratios (95% confidence intervals) for incident diabetes from logistic regression analyses.

Model 1 is adjusted for age and sex; model 2 is adjusted for age, sex, systolic blood pressure, antihypertensive treatment, waist circumference, plasma levels of triglycerides, fasting whole-blood glucose and HDL at baseline. *n*=3,068 (403 cases and 2,665 controls).
